# Effects of exercise interventions on fundamental motor skills in children and adolescents with autism spectrum disorder: a meta-analysis with meta-regression analyses of randomized controlled trials

**DOI:** 10.3389/fpsyt.2026.1954728

**Published:** 2026-09-30

**Authors:** Jiangdi Su, Liang Li, Yuchen Wang, Tonggang Fan

**Affiliations:** 1 College of Wushu, Shanghai University of Sport, Shanghai, China; 2 Hebei Sport University, Shijiazhuang, Hebei, China

**Keywords:** autism spectrum disorder, exercise intervention, locomotor skills, object control skills, stability skills

## Abstract

**Purpose:**

The objective of this study was to systematically evaluate the effects of exercise interventions on fundamental motor skills (FMS) in children and adolescents with autism spectrum disorder (ASD) and determine whether distinct FMS domains demonstrate differential responses to exercise dosage and intervention characteristics.

**Methods:**

A comprehensive search of PubMed, the Cochrane Library, Embase, and Web of Science up to July 2026 identified 31 RCTs. Standardized mean differences were calculated to evaluate the effects of exercise interventions on object control, locomotor, and stability skills. Subgroup and meta-regression analyses examined the effects of intervention characteristics and exercise dosage, while sensitivity analyses and risk of bias assessments evaluated the robustness of the findings.

**Results:**

Exercise interventions significantly improved all three dimensions of FMS in children and adolescents with ASD: Object control skills (OCS) (Hedges’ *g* = 1.13, 95% CI: 0.67 to 1.59), Locomotor skills (LMS) (Hedges’ *g* = 1.19, 95% CI: 0.87 to 1.51), and Stability skills (SS) (Hedges’ *g* = 1.08, 95% CI: 0.68 to 1.49). Further analyses revealed domain-specific responses to exercise dosage. Longer exercise session time was associated with greater improvements in OCS, supported by both subgroup analyses and meta-regression. For LMS, greater effects were observed with three sessions per week and interventions lasting ≤ 10 weeks in subgroup analyses, although these findings were not confirmed by meta-regression. No significant dose-related associations were identified for SS.

**Conclusion:**

Exercise interventions improve OCS, LMS, and SS in children and adolescents with ASD. Different FMS domains may show distinct responses to exercise dosage, suggesting that future exercise programs may need to consider the specific characteristics of targeted motor domains.

**Systematic review registration:**

https://www.crd.york.ac.uk/PROSPERO/view/CRD420251139963, identifier CRD420251139963.

## 1 Introduction

Autism Spectrum Disorder (ASD) is a complex neurodevelopmental condition, characterized by core features including difficulties in social communication and interaction, as well as restricted, repetitive patterns of behavior, interests, or activities (1). Recent epidemiological evidence has shown that the prevalence of ASD among 8-year-old children in the United States reached 32.2 per 1,000 (approximately 1 in 31) by 2022, marking a notable increase compared to that in previous years. Similar upward trends have been documented globally (2). This creates increasingly substantial challenges for children and adolescents with ASD in education, social integration, and daily living. Beyond core symptoms, individuals with ASD frequently experience co-occurring difficulties such as sensory processing issues, intellectual disabilities, and motor impairments. These additional challenges may further disrupt developmental trajectories and reduce quality of life.

Accumulating evidence indicates that motor impairment is a salient characteristic of children with ASD (3). Approximately four out of five children with ASD demonstrate developmental delays or atypical motor patterns (4), with deficits in fundamental motor skills (FMS) being the most pronounced.

FMS are widely recognized as essential to children’s motor development, forming the foundation for acquiring complex movement patterns and maintaining lifelong physical activity (5). They are generally categorized into three domains: locomotor skills (LMS) (e.g., running, jumping), object control skills (OCS) (e.g., throwing, catching), and stability skills (SS) (e.g., balancing, turning). Children with ASD often display deficits in these areas, which may be associated with neurodevelopmental delays and atypical brain functioning (6, 7), with such difficulties frequently extending into adolescence (8). Beyond limiting motor competence, these impairments restrict opportunities for regular physical activity and increase the susceptibility to social withdrawal, sedentary lifestyles, obesity, and mental health challenges (9), thereby reinforcing a detrimental cycle of motor deficits, reduced participation, and heightened health risks.

Among the strategies used to improve motor competence in children and adolescents with ASD, exercise interventions are increasingly recognized as an effective approach because of their diverse formats, ease of implementation, and minimal adverse effects. Exercise interventions may improve motor skills by promoting neuroplasticity, enhancing functional brain connectivity, and modulating neurotransmitter levels (10–12). Interventions such as equestrian sports, swimming, and judo can significantly improve motor coordination, balance, and skills in children with ASD (13–15). However, some studies failed to observe any significant effects (16), indicating that the efficacy of exercise interventions varies and may be closely related to factors such as intervention type, duration, frequency, intensity, and individual characteristics.

In recent years, systematic reviews and meta-analyses have consistently confirmed the beneficial effects of exercise interventions on FMS in children with ASD (17–19). Previous studies have demonstrated that exercise interventions can improve multiple FMS domains, including LMS, OCS, and SS. Moreover, increasing attention has been given to the potential influence of intervention characteristics and exercise dosage on motor outcomes. For example, Ji et al. (18) and Ye et al. (19) explored the effects of exercise modality, intervention frequency and duration on FMS improvements, while more recent studies have further applied subgroup analyses and meta-regression approaches to identify potential moderators, including dosage-related factors (20–22). However, although previous studies have explored dosage-related factors such as intervention duration and frequency, the optimal exercise dosage for improving FMS remains uncertain. Furthermore, it remains unclear whether different FMS domains exhibit distinct dose–response relationships with exercise dosage.

To address the limitations of previous reviews, this updated meta-analysis examined whether exercise dosage has differential effects across FMS domains in children and adolescents with ASD. To provide a more comprehensive evidence base, newly published studies were incorporated, and the effects of exercise interventions were evaluated separately across three FMS domains: OCS, LMS, and SS. Based on these domain-specific analyses, subgroup analyses compared the effects of different intervention characteristics and dosage categories, whereas meta-regression analyses further explored potential dose–response relationships between exercise parameters and FMS outcomes. By integrating these findings, this study clarifies domain-specific dosage patterns and provides more targeted evidence to inform exercise prescription for children and adolescents with ASD.

## 2 Methods

### 2.1 Design

This meta-analysis was conducted according to the PRISMA guidelines (23) and the Cochrane Handbook for Systematic Reviews of Interventions (24). The protocol was registered in PROSPERO (CRD420251139963).

### 2.2 Search strategy

A systematic search was conducted in PubMed, the Cochrane Library, Embase, and Web of Science in two phases. The first retrieval covered database inception to October 2025, and the second retrieval covered October 2025 to July 2026. The same search strategy was used in both phases, with only the search period updated in the second retrieval. The updated search was conducted to ensure that the most recent eligible studies were included and that the available evidence was comprehensively covered. Subject headings and free-text terms were combined under the PICOS framework: (Population) AND (Intervention) AND (Outcome) AND (Child/Adolescent filter). Reference lists and citation records of relevant reviews were also screened to identify additional eligible studies. The complete database strategies are provided in the **Supplementary Material**.

### 2.3 Inclusion criteria

This study included RCTs. Participants were children and adolescents under 18 years of age with a clinically confirmed diagnosis of ASD, irrespective of sex or ethnicity. The intervention group received a structured physical activity program, whereas the control group received no intervention, served as a waitlist control, received routine care, or maintained their usual daily activities. Outcome measures required standardized, validated motor skills assessment tools such as TGMD-2, TGMD-3, BOT-2, and MABC-2. Studies were required to report complete pre- and post-intervention data, including sample size, mean scores, and standard deviations.

The exclusion criteria were as follows: non-randomized controlled trials, animal studies, conference abstracts lacking full texts, duplicate publications, studies with incomplete outcome data or missing key outcome measures, and reports for which the full text could not be obtained.

### 2.4 Data extraction and quality assessment

#### 2.4.1 Data extraction process

Two reviewers (LL and WY) independently screened the literature, extracted relevant data, and verified the results against the predefined eligibility criteria. Any discrepancies were addressed through discussion and, when necessary, resolved with input from a third reviewer. The extracted information included: (1) general study characteristics such as title, first author, publication year, and country; (2) participant demographics and baseline data (e.g., age, sample size); and (3) details of interventions in both experimental and control groups, including intervention type, intervention frequency, intervention duration, exercise session duration, and outcome measures and corresponding results.

In addition, based on previous studies (25–29) and the intervention descriptions of the included studies, exercise Interventions were classified into five categories. The definitions of each category are presented in **Table 1**.

**Table 1:** Operational definitions of treatment nodes included in the meta-analysis.

Node	Operational definition
Exergaming	Exergaming was defined as interactive, digital game–based physical activity in which participants are required to perform bodily movements as input for gameplay or to complete game-based exercise tasks, such as Nintendo Wii and Xbox Kinect (29).
Fundamental movement skill training (FMST)	Fundamental movement skill training was defined as structured exercise primarily designed to develop or improve fundamental movement skills through targeted practice of basic movements, such as running, jumping, hopping, throwing, catching, kicking, striking, and balancing (27).
Aquatic exercise (AE)	Aquatic exercise was defined as structured physical exercise performed in a water-based environment (28); examples include aquatic training, adapted aquatic exercise, Halliwick-based programs, and swimming training.
Mind–body exercise (MBE)	Mind–body exercise was defined as exercise that integrates controlled physical movements with breathing regulation and attentional or meditative components, emphasizing coordinated involvement of the body and mind, such as Tai Chi, Pilates, and yoga (25, 26).
Multicomponent physical activity (MPA)	Multicomponent physical activity was defined as a structured exercise intervention integrating two or more distinct training components, including aerobic exercise, strength, balance, coordination, agility, flexibility, and cognitive engagement.

#### 2.4.2 Quality assessment

The Physiotherapy Evidence Database (PEDro) scale was used to assess the methodological quality of the included RCTs. The scale consists of 11 items, with the first item (eligibility criteria) excluded from the total score. The remaining 10 items assess random allocation, concealed allocation, baseline comparability, blinding of participants, blinding of therapists, blinding of assessors, adequate follow-up, intention-to-treat analysis, between-group comparisons, and point estimates and measures of variability. Each item was scored as 1 (satisfied) or 0 (not satisfied), with a maximum score of 10. Scores of <4, 4–5, 6–8, and 9–10 were classified as poor, fair, good, and excellent quality, respectively (30). Two reviewers independently assessed study quality, and disagreements were resolved by discussion with a third reviewer.

### 2.5 Data analysis

Owing to the diversity of motor skill assessment instruments across studies, Hedges’ *g* was applied as the effect-size metric. This metric enabled a comparison of the effects of exercise interventions on three core domains of motor performance in children with ASD: OCS, LMS, and SS. Corresponding 95% confidence intervals (CIs) were calculated to assess the precision and statistical significance of the pooled estimates. Statistical analyses were performed using R with the metafor package. To account for potential heterogeneity arising from differences in participant characteristics and intervention protocols, heterogeneity was quantified using the *I*² statistic. When *I*² exceeded 50%, indicating substantial between-study heterogeneity, a random-effects model was applied. Given the inconsistency in how overall FMS were defined and measured across assessment instruments, pooled analyses of global FMS were not conducted. Instead, separate meta-analyses were performed for OCS, LMS, and SS. For multi-arm trials, each intervention group was compared independently with the shared control group. To avoid double-counting, the control group sample size was divided equally across the intervention comparisons. Additionally, to assess the influence of studies with lower methodological quality, sensitivity analyses were conducted by temporarily excluding studies with PEDro scores of 4–5. The pooled effects were then recalculated using the same random-effects model.

### 2.6 Subgroup analyses

Building upon previous meta-analytic findings, we conducted multidimensional subgroup analyses to systematically examine whether intervention characteristics and measurement instruments influenced the effects of exercise interventions on FMS in children with ASD. Specifically, the analyses focused on three key motor domains: OCS, LMS, and SS. Intervention characteristics were explored across several dimensions. First, intervention type was categorized into multicomponent physical activity (MPA), fundamental movement skill training (FMST), aquatic exercise (AE), mind–body exercise (MBE), and exergaming according to the primary components and characteristics of each intervention program. Second, intervention frequency was classified based on weekly training sessions as 1–2 sessions/week, 3 sessions/week, and 4–5 sessions/week, based on previous meta-analytic classifications and the frequency distribution of the included studies (31, 32). Third, exercise session duration was categorized as < 60 minutes and ≥ 60 minutes to examine its potential influence on intervention effects, with reference to previous meta-analyses and the distribution of the included studies (18, 33). Fourth, intervention duration was grouped into ≤ 10 weeks and > 10 weeks to assess whether program length influenced the intervention effects, based on previous meta-analytic classifications and the distribution of the included studies (34). Fifth, participant age was categorized into 3–7 years and 7–12 years based on age classifications adopted in previous studies and the age distribution of participants across the included studies (35). Finally, subgroup analyses based on measurement instruments were performed to assess potential measurement-related heterogeneity, with included assessment instruments comprising the Test of Gross Motor Development (TGMD), Bruininks–Oseretsky Test of Motor Proficiency (BOT), Movement Assessment Battery for Children (MABC-2), One-Leg Standing test (OLS), Berg Balance Scale (BBS), and center of pressure (COP) measures.

### 2.7 Publication bias analysis

To assess the risk of publication bias, this study first conducted a preliminary assessment by plotting funnel plots using R. When funnel plots exhibited marked asymmetry, Egger’s regression bias test was used to quantitatively evaluate small-sample effects and potential publication bias. Subsequently, to investigate the specific impact of bias on the overall effect size estimation, the trim-and-fill method was applied. This method was used to estimate the potential number of missing studies and obtain bias-adjusted pooled effect estimates.

## 3 Results

### 3.1 Literature search and screening process

**Figure 1** illustrates the detailed workflow of literature retrieval and study selection. The first retrieval identified 1,360 records from Web of Science (n = 643), PubMed (n = 182), Embase (n = 407), Cochrane Library (n = 125), and other sources (n = 3). After removing 486 duplicate records, 874 records were screened based on titles and abstracts, and 815 were excluded. Of the 59 reports assessed for eligibility, 34 were excluded due to non-RCT design (n = 16), incomplete data (n = 9), or unavailable full texts (n = 9). Finally, 25 studies were included from the first retrieval. The second retrieval, conducted from October 2025 to July 2026, identified 224 additional records from Web of Science (n = 97), PubMed (n = 34), Embase (n = 50), and Cochrane Library (n = 43). After removing 84 duplicate records, 140 records were screened, and 117 were excluded based on titles and abstracts. Among the 23 full-text articles assessed for eligibility, 17 were excluded due to ineligible outcomes (n = 5), non-randomized designs (n = 6), incomplete data (n = 2), ineligible age range (n = 1), or unsuitable control groups (n = 3). 6 additional studies were included from the updated retrieval. After merging the two retrievals, a total of 31 randomized controlled trials were included in the final quantitative synthesis (**Figure 1**).

**Figure 1:**
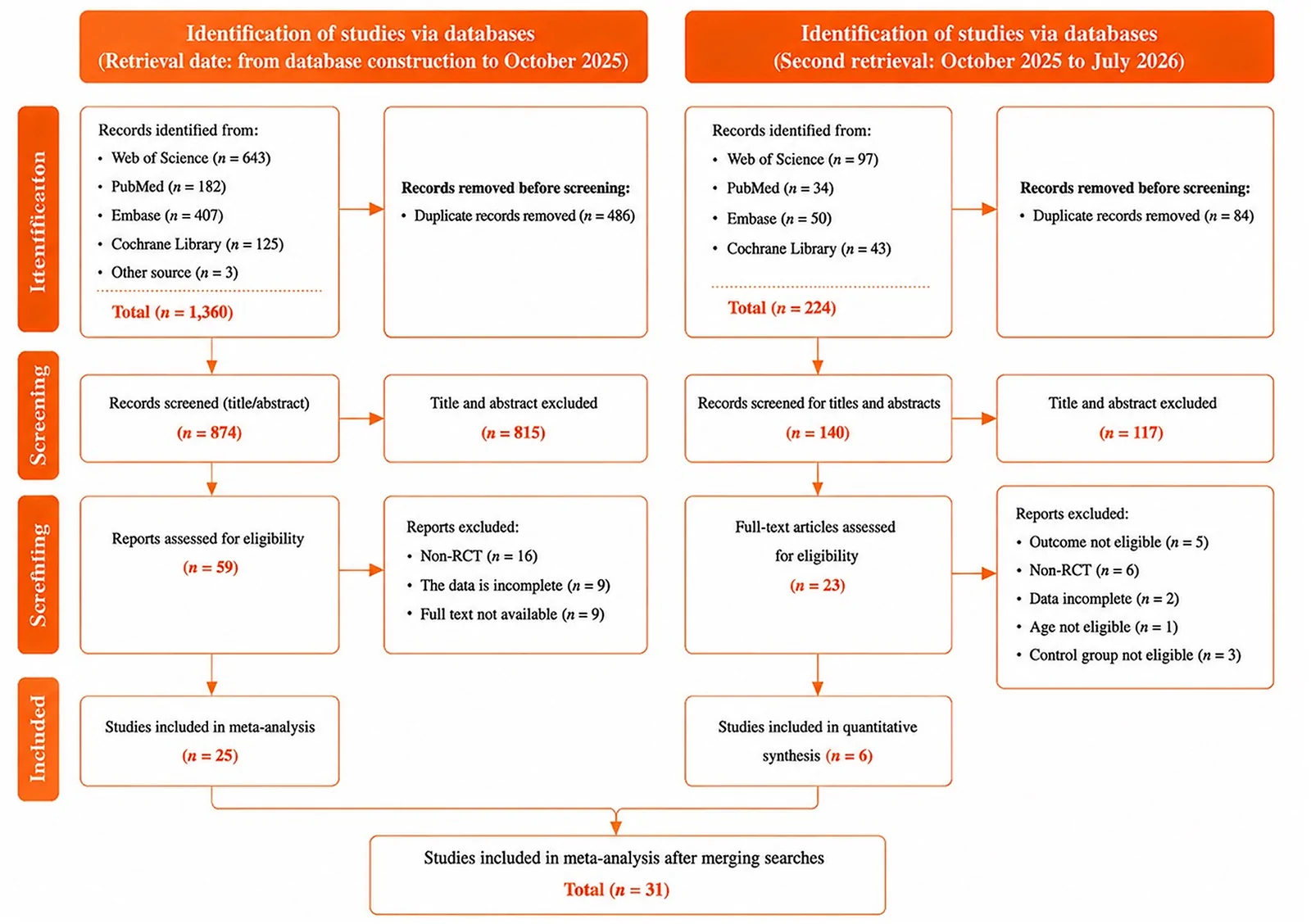
PRISMA flow diagram for studies retrieved through the electronic search and selection processes.

### 3.2 Characteristics of included studies

This meta-analysis included 31 studies conducted across Asia, Europe, North America, Africa, and Australia (27, 36–65). A total of 880 children and adolescents with ASD were included. Participants were primarily children aged approximately 4.3–12.8 years. The interventions included FMST, MPA, MBE, AE, exergaming, and other structured physical activity programs. Intervention protocols varied considerably, with frequencies ranging from 1 to 5 sessions/week, intervention durations ranging from 2 to 18 weeks, and session durations ranging from 30 to 80 minutes. Detailed study characteristics are presented in **Table 2**.

**Table 2:** Characteristics of the included studies.

Study (year, country)	Participants characteristics			Intervention characteristics					Outcome	Follow-up time
	Age (EG,CG)	Diagnostic methods	Sample size (EG,CG)	Type	Time	Frequency	Duration	Intensity		
Bremer et al. (2015, Canada) (36)	4.30 ± 0.25/4.33 ± 0.22	Clinical diagnosis	EG 5/CG 4	FMST	60	1	12	NR	PDMS-2	6 weeks
Pan et al. (2017, Taiwan) (37)	9.68 ± 1.61/8.49 ± 1.76	DSM-IV-TR	EG 11/CG 11	Table tennis	70	2	12	NR	BOT-2	12 weeks
Ghayour Najafabadi et al. (2018, Iran) (38)	7.08 ± 2.06/5.07 ± 2.23	DSM-IV-TR	EG 12/CG 14	SPARK	40	3	12	NR	BOTMP	NR
Arabi et al. (2019, Iran) (39)	8.4 ± 2.01/8.44 ± 1.94	DSM-5	EG1 15/CG 15	SPARK	60	3	10	NR	TGMD-2	8 weeks
Sarabzadeh et al. (2019, Iran) (40)	8.88 ± 1.76/8.22 ± 1.92	Clinical diagnosis	EG 9/CG 9	Tai Chi Chuan	60	3	6	NR	MABC-2	NR
Ansari et al. (2021, Iran) (41)	10.6 ± 2.5/10.8 ± 2.14	DSM-5	EG1 10/EG2 10/CG 10	Aquatic exercise/Kata	60	2	10	NR	Modified Stork Standing Test	NR
Hassani et al. (2020, Iran) (43)	9.10 ± 0.87/8.55 ± 0.68/8.70 ± 0.70	DSM-5	EG1 10/EG2 11/CG 9	SPARK/ICPL	60	2	8	NR	BOT-2	NR
Rafiei Milajerdi et al. (2021, Iran) (42)	7.95 – 1.60/8.15 – 1.50/8.45 – 1.43	ADOS-2	EG1 20/EG2 20/CG 20	SPARK/Kinect	35	3	8	Light-to-moderate physical activity	MABC-2	NR
Marzouki et al. (2022, Tunisia) (44)	6.3 ± 0.5/6.4 ± 0.5	DSM-5	EG1 8/EG2 8/CG 6	TAT/GAT	60	2	8	NR	TGMD-2	NR
Shanker and Pradhan (2022, India) (45)	9.77 ± 2.63/9.61 ± 1.931.36	Clinical diagnosis	EG 23/CG 20	Yoga	45	5	12	NR	BOT-2	NR
Zhao et al. (2022, China) (46)	6.0 ± 1.7/5.8 ± 1.5	DSM-5	EG 26/CG 27	Equestrian	60	2	12	NR	TGMD-3	NR
Deng et al. (2023, China) (47)	6.56 ± 1.42/6.22 ± 0.97	Clinical diagnosis	EG 9/CG 9	Sensory integration training	60	3	8	NR	Sharpened Romberg Test (Footscan)	NR
Faraji et al. (2023, Iran) (48)	7.26 ± 1.54/8.20 ± 1.38	DSM-5	EG 20/CG 20	Aquatic therapy	45	3	8	NR	BOTMP-2	NR
Haghighi et al. (2023, Iran) (49)	9.00 ± 1.31/8.13 ± 1.36	Clinical diagnosis	EG 8/CG 8	Combined physical training	60-80	3	8	NR	Stork Test; agility T-test	NR
Lindor et al. (2023, Australia) (50)	9.00 ± 1.47/9.08 ± 1.44	Clinical diagnosis	EG 8/CG 9	Dance	60	1	10	NR	MABC-2	NR
Cui and Wang (2024, China) (65)	8.08 ± 1.97/8.25 ± 1.28	Clinical diagnosis	EG 12/CG 12	Dance	80	3	12	NR	TGMD-3	NR
Hatipoglu Ozcan et al. (2024, Turkey) (54)	4.84 ± 0.73/4.69 ± 0.75	Clinical diagnosis	EG 17/CG 17	MIP	60	2	12	NR	PDMS-2	NR
Ju et al. (2024, China) (51)	11.11 ± 2.52/12.75 ± 2.31	Clinical diagnosis	EG 9/CG 8	Yoga intervention	45-50	3	8	NR	MABC-2	4 weeks
Kanzari et al. (2025, Tunisia) (55)	7.8 ± 1.94/8.4 ± 3.03	DSM-5-TR	EG 10/CG 11	Music- and movement-based intervention	45	3	8	NR	BOT-2	NR
Pan et al. (2025, Taiwan) (58)	6.35 ± 1.78/5.91 ± 1.87	Clinical diagnosis	EG 10/CG 10	FMST	60	2	12	NR	TGMD-2	12 weeks
Falivene et al. (2025, Italy) (52)	9.14 ± 1.85/10.75 ± 1.71	DSM-5; ADOS-2	EG 10/CG 10	Exergame	45	2	5	NR	MABC-2	NR
Hashempour Alooche et al. (2025, Iran) (53)	9.46 ± 1.94/8.5 ± 1.84	Clinical diagnosis	EG 15/CG 15	Physical activity training	45	3	8	NR	Force-plate COP;	NR
Pan et al. (2025, Taiwan/USA collaboration) (57)	6.23 ± 1.88/6.30 ± 1.58	DSM-5-TR	EG 23/CG 23	FMST	60	2	12	NR	TGMD-2	NR
Qi et al. (2025, China) (59)	6.52 ± 2.19/6.40 ± 2.30	Clinical diagnosis	EG 23/CG 20	BCTP	45	5	12	128–148 beats/min	MABC-2;	NR
Wang et al. (2025, China) (27)	7.75 ± 1.65/7.63 ± 1.58	Clinical diagnosis	EG 12/CG 10	FMST	45	4	18	NR	TGMD-3	NR
Aly et al. (2026, Egypt) (61)	9.50 ± 2.56/9.25 ± 2.66	DSM-5	EG 8/CG 8	Sensory-motor exercise	45	4	16	NR	Berg Balance Scale	NR
Kruger et al. (2026, Brazil) (62)	8–10 years	NR	EG 19/CG 20	Physical exercise program	50	3	16	NR	TGMD-2	NR
Liu et al. (2026, China) (63)	7.25 ± 0.41/7.17 ± 0.40	DSM-5	EG 6/CG 6	Halliwick aquatic exercise	60-70	3	12	NR	One-leg stance;	4 weeks
Luo et al. (2026, China) (64)	11.28 ± 1.54/12.04 ± 1.51	DSM-5	EG 25/CG 25	Trampoline training	30	3	8	NR	One-Leg Stance	NR
Luo et al. (2025, Thailand/China) (56)	10.17 ± 1.17/10.17 ± 1.17	Clinical diagnosis	EG 6/CG 6	Trampoline training	30	3	2	NR	One-Leg Stance	NR
Wu et al. (2026, China) (60)	8.4 ± 1.31/8.05 ± 1.27	ADOS-2	EG 20/CG 20	Exergame	45	3	12	NR	TGMD-2	NR

TGMD-2, Test of Gross Motor Development – Second Edition; TGMD-3, Test of Gross Motor Development – Third Edition; BOT-2, Bruininks-Oseretsky Test of Motor Proficiency – Second Edition; BOTMP, Bruininks-Oseretsky Test of Motor Proficiency; MABC-2, Movement Assessment Battery for Children – Second Edition; PDMS-2, Preschool Motor Development Scale – Second Edition; SPARK, SPARK Motor Training Program; ICPL, I Can have Physical Literacy; TAT, Technical Aquatic Activities Program; GAT, Game-based Aquatic Activities Program; MIP, Motor Intervention Program; BCTP, Ball Combination Training Program; FMST, Fundamental Movement Skill Training

### 3.3 Quality assessment

The 31 articles included in the meta-analysis were assessed using the PEDro scale and received scores ranging from 4 to 8, with most studies scoring 6 points, indicating an overall moderate-to-good methodological quality. Seven trials were rated as moderate quality (scores 4–5), whereas 24 trials were classified as good quality (scores 6–8) (see **Table 3**).

**Table 3:** Methodological quality of included RCTs assessed by the PEDro scale.

Study	Randomization allocation	Allocation concealment	Similar at baseline	Subject blinded	Therapist blinded	Assessor blinded	Dropout rate	Intention-to-treat analysis	Between-group comparison	Point measures	Total score	Quality rating
Bremer et al. (36)	1	0	1	0	0	0	1	0	1	1	**5**	**Moderate**
Pan et al. (37)	1	0	1	0	0	0	1	1	1	1	**6**	**Good**
Ghayour Najafabadi et al. (38)	1	0	1	0	0	1	1	1	1	1	**7**	**Good**
Arabi et al. (39)	1	0	1	0	0	0	1	1	1	1	**6**	**Good**
Sarabzadeh et al. (40)	1	0	1	0	0	0	1	1	1	1	**6**	**Good**
Ansari et al. (41)	1	0	1	0	0	0	1	1	1	1	**6**	**Good**
Hassani et al. (43)	1	0	1	0	0	1	1	1	1	1	**7**	**Good**
Rafiei Milajerdi et al. (42)	1	0	1	0	0	0	1	1	1	1	**6**	**Good**
Marzouki et al. (44)	1	0	1	0	0	0	0	0	1	1	**4**	**Moderate**
Shanker and Pradhan (45)	1	0	1	0	0	0	1	1	1	1	**6**	**Good**
Zhao et al. (46)	1	0	1	0	0	0	0	0	1	1	**4**	**Moderate**
Deng et al. (47)	1	0	1	0	0	0	1	0	1	1	**6**	**Good**
Faraji et al. (48)	1	0	1	0	0	0	1	1	1	1	**6**	**Good**
Haghighi et al. (49)	1	0	1	0	0	1	1	1	1	1	**7**	**Good**
Lindor et al. (50)	1	1	1	0	0	1	0	0	1	1	**6**	**Good**
Cui and Wang (65)	1	0	1	0	0	0	1	1	1	1	**6**	**Good**
Hatipoglu Ozcan et al. (54)	1	0	1	0	0	0	1	1	1	1	**6**	**Good**
Ju et al. (51)	1	1	1	0	0	0	1	1	1	1	**7**	**Good**
Kanzari et al. (55)	1	0	1	0	0	0	1	1	1	1	**7**	**Good**
Pan et al. (58)	1	0	1	0	0	0	1	1	1	1	**6**	**Good**
Falivene et al. (52)	1	1	1	0	0	0	1	0	1	1	**6**	**Good**
Hashempour Alooche et al. (53)	1	0	1	0	0	0	1	1	1	1	**6**	**Good**
Pan et al. (57)	1	0	1	0	0	0	1	1	1	1	**6**	**Good**
Qi et al. (59)	1	0	1	0	0	0	0	0	1	1	**4**	**Moderate**
Wang et al. (27)	1	0	1	0	0	0	1	0	1	1	**5**	**Moderate**
Aly et al. (61)	1	1	1	0	0	1	0	0	1	1	**5**	**Moderate**
Kruger et al. (62)	1	0	1	0	0	0	0	0	1	1	**4**	**Moderate**
Liu et al. (63)	1	1	1	0	0	1	1	1	1	1	**8**	**Good**
Luo et al. (64, China)	1	1	1	0	0	1	1	1	1	1	**8**	**Good**
Luo et al. (56, Thailand/China)	1	0	1	0	0	0	1	1	1	1	**6**	**Good**
Wu et al. (60, China)	1	0	1	0	0	1	1	0	1	1	**6**	**Good**

All studies applied random allocation and demonstrated baseline comparability between groups. In addition, all trials reported between-group comparisons for the main outcomes, together with corresponding point estimates and measures of variability, supporting the reliability of the reported findings. However, allocation concealment was reported in only seven studies, and none of the trials implemented participant or therapist blinding. Assessor blinding was reported in eight studies, indicating potential risks of selection and detection bias. Most studies adequately addressed dropout rates (28/31) and applied intention-to-treat analyses (23/31).

### 3.4 Meta-analysis results

#### 3.4.1 Effects of exercise interventions on OCS

The OCS meta-analysis included 20 studies contributing 23 effect-size estimates and involving 604 participants across the experimental and control groups. Heterogeneity analysis (**Figure 2**) revealed substantial between-study heterogeneity (*τ*² = 0.95, *I*² = 83.23%, *Q*(22) = 90.91, *p* <.001), necessitating the use of a random-effects model for synthesis (**Figure 2**). Results indicated that exercise interventions significantly improved OCS in children and adolescents with ASD (Hedges’ *g* = 1.13, 95% CI: 0.67–1.59, *p* <.001), with the difference being statistically significant. Notably, some studies (e.g., 35, 38) showed relatively large effect sizes, which may have influenced the pooled estimate.

**Figure 2:**
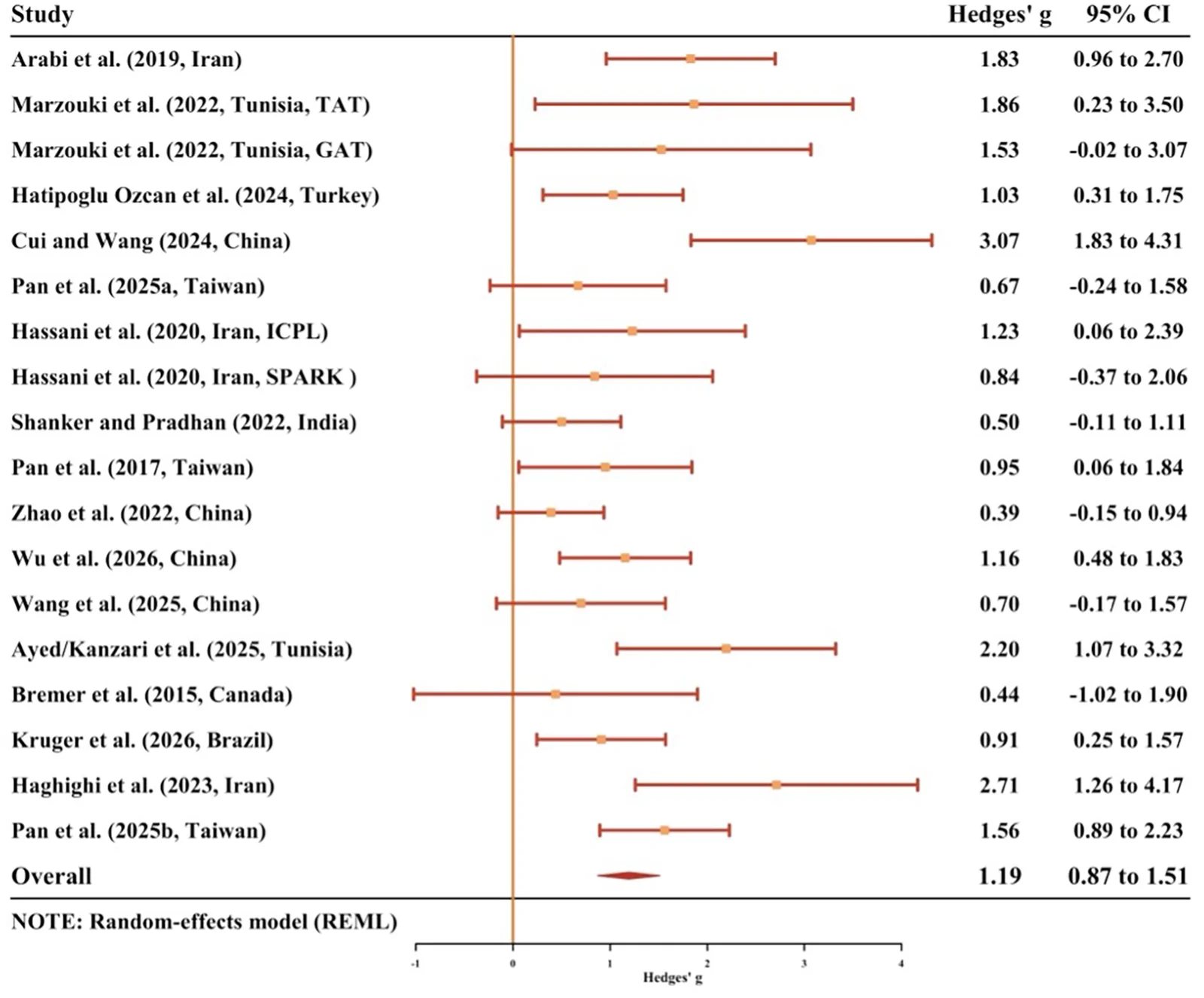
Forest plot of the OCS.

#### 3.4.2 Effects of exercise interventions on LMS

The LMS meta-analysis included 16 studies contributing 18 effect-size estimates and involving 470 participants across the experimental and control groups. Heterogeneity analysis (**Figure 3**) revealed significant between-study heterogeneity (*τ*² = 0.23, *I*² = 54.33%, *Q*(17) = 37.10, *p* = .003), and a random-effects model was applied (**Figure 3**). Results indicated that exercise interventions significantly improved LMS in children and adolescents with ASD (Hedges’ *g* = 1.19, 95% CI: 0.87–1.51, *p* <.001), with the difference being statistically significant. Notably, some studies (e.g., 44, 60) showed relatively larger effect sizes, which may have contributed to the observed heterogeneity.

**Figure 3:**
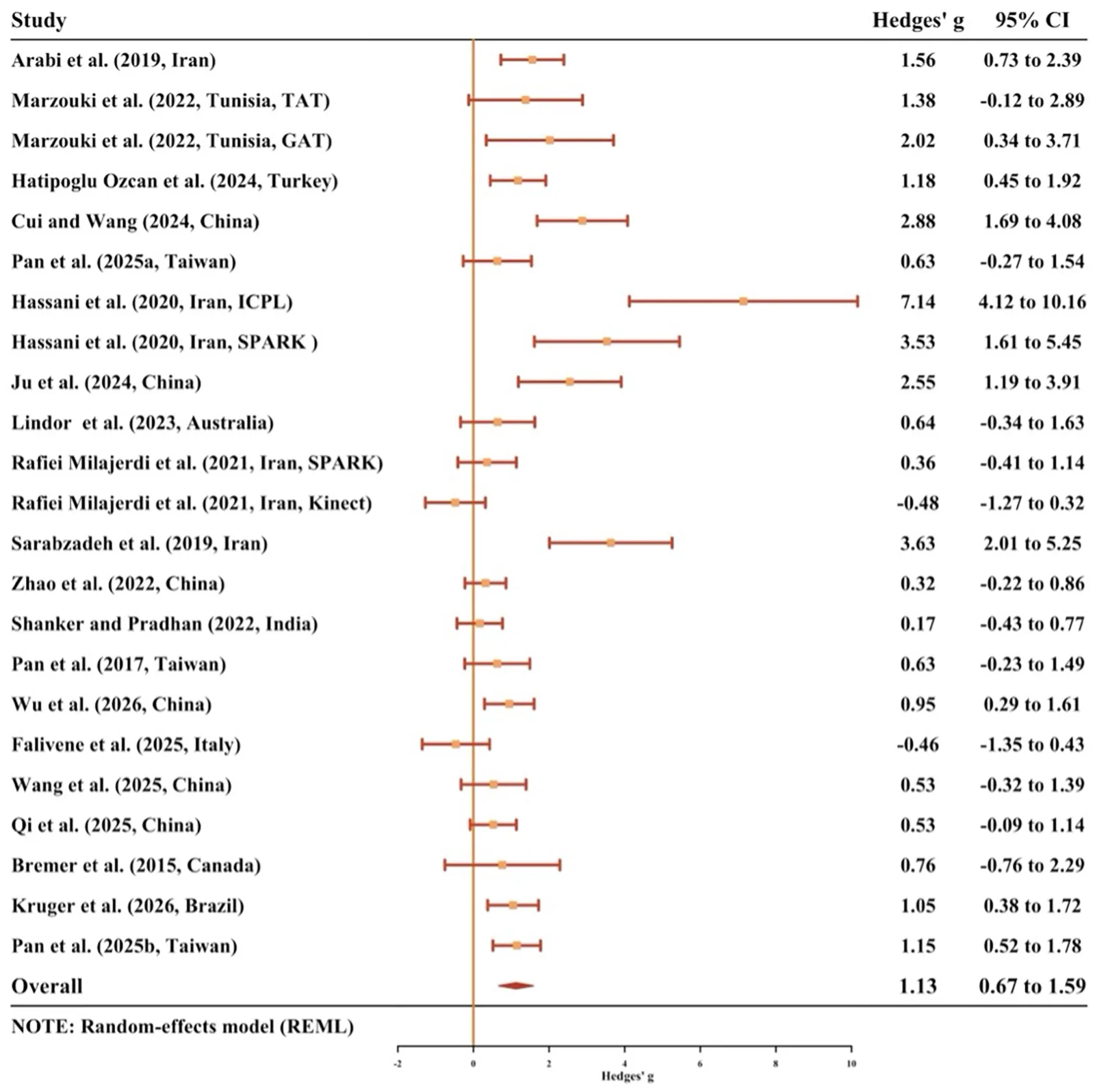
Forest plot of the LMS.

#### 3.4.3 Effects of exercise interventions on SS

The SS meta-analysis included 20 studies contributing 23 effect-size estimates and involving 522 participants across the experimental and control groups. Heterogeneity analysis (**Figure 4**) revealed significant between-study heterogeneity (*τ*² = 0.71, *I*² = 76.05%, *Q*(22) = 83.93, *p* <.001), supporting the use of a random-effects model (**Figure 4**). The pooled results indicated that exercise interventions significantly improved SS in children and adolescents with ASD (Hedges’ *g* = 1.08, 95% CI: 0.68–1.49, *p* <.001). Notably, Sarabzadeh et al. (35)Aly et al. (56), and Liu et al. (58) demonstrated relatively larger effect sizes, which may have contributed to the observed heterogeneity.

**Figure 4:**
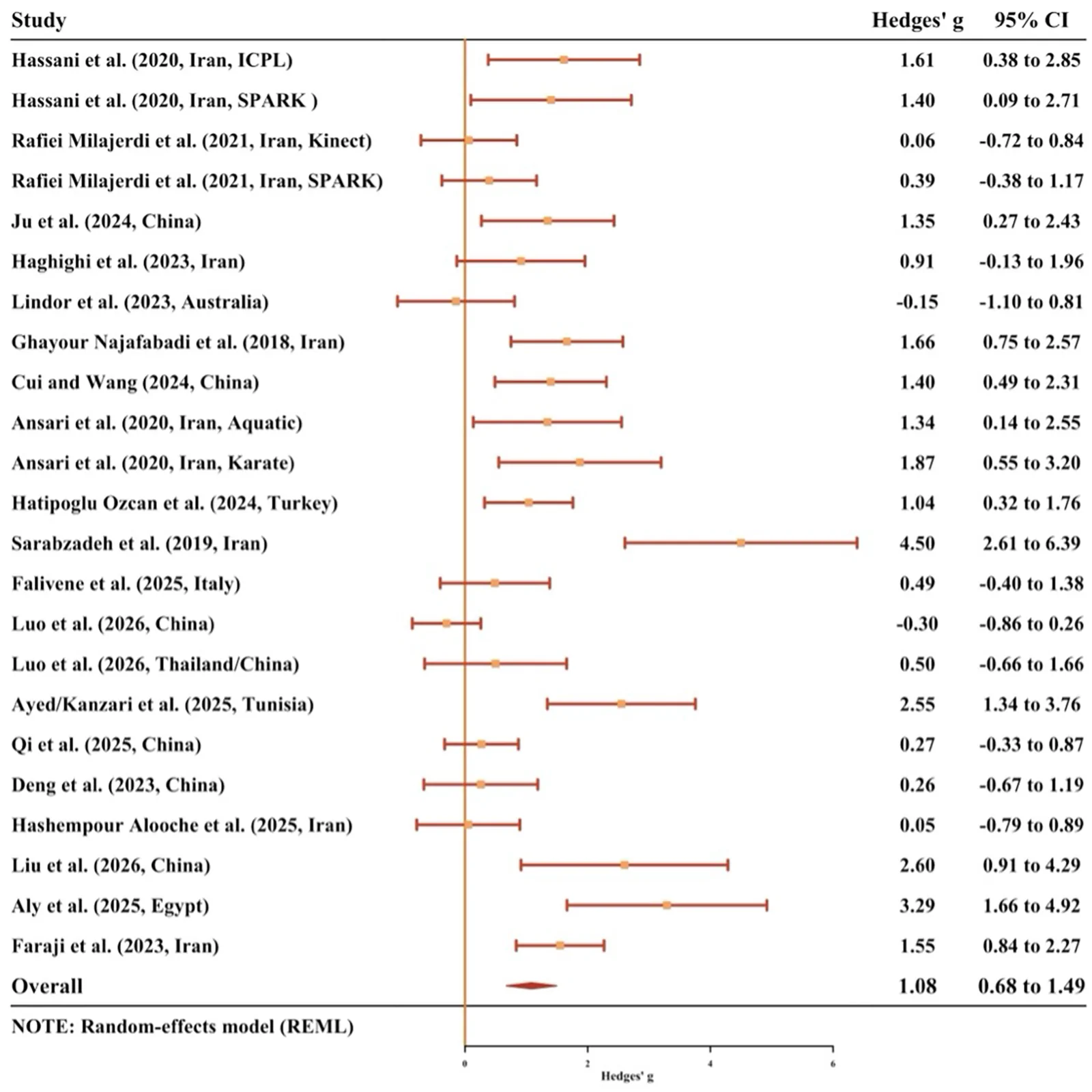
Forest plot of the SS.

### 3.5 Subgroup analyses

#### 3.5.1 OCS subgroup analysis

Subgroup analyses indicated that exercise interventions tended to improve OCS across different intervention parameters and measurement approaches (**Figure 5**), although significant effects were not observed in all subgroups. Further moderator analyses showed that only exercise session duration significantly moderated between-group differences, suggesting that session duration may be associated with variations in the magnitude of OCS improvements.

**Figure 5:**
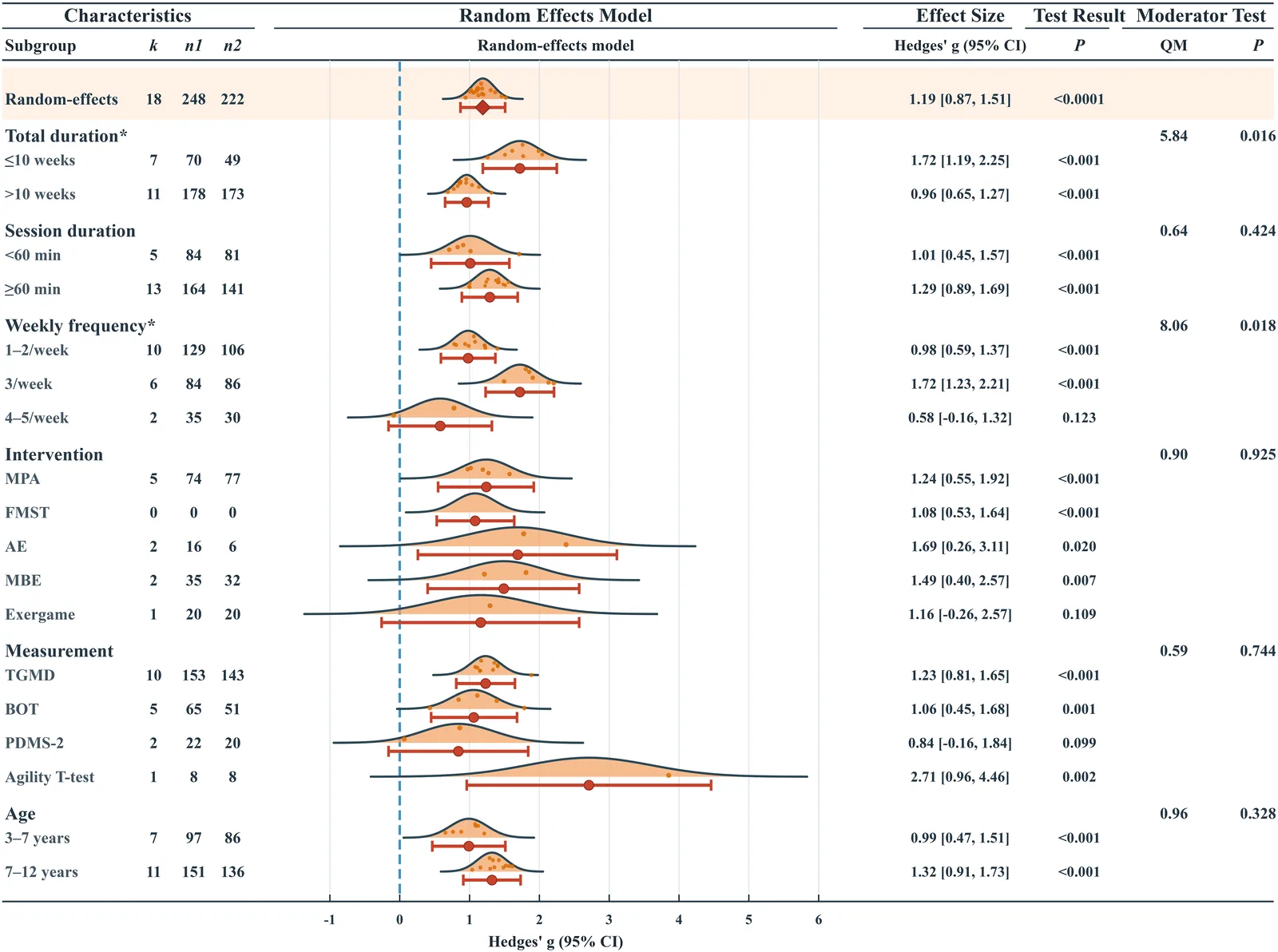
Subgroup analysis of OCS. Note. n1 = number of participants in the intervention group; n2 = number of participants in the control group.

Intervention frequency was not a significant moderator of OCS (*QM*(2) = 2.06, *p* = .357). Significant effects were observed for 1–2 sessions/week (Hedges’ *g* = 1.15, 95% CI: 0.51–1.78) and 3 sessions/week (Hedges’ *g* = 1.47, 95% CI: 0.64–2.31), whereas 4–5 sessions/week did not reach statistical significance (Hedges’ *g* = 0.41, 95% CI: -0.79–1.60).

Intervention duration did not significantly moderate the effects on OCS (*QM*(1) = 1.68, *p* = .195). Both ≤ 10 weeks (Hedges’ *g* = 1.50, 95% CI: 0.78–2.22) and > 10 weeks (Hedges’ *g* = 0.87, 95% CI: 0.25–1.49) intervention programs showed significant improvements in OCS.

Exercise session duration was the only significant moderator of OCS (*QM*(1) = 5.88, *p* = .015). Sessions lasting ≥ 60 min showed a significant effect (Hedges’ *g* = 1.54, 95% CI: 0.99–2.09), whereas sessions lasting < 60 min did not reach statistical significance (Hedges’ *g* = 0.53, 95% CI: -0.09–1.14, *p* = .093).

Intervention type did not significantly explain between-subgroup differences in OCS (*QM*(4) = 6.91, *p* = .141). Significant within-group effects were found for FMST (Hedges’ *g* = 1.37, 95% CI: 0.60–2.14) and MBE (Hedges’ *g* = 1.77, 95% CI: 0.81–2.72). In contrast, MPA (Hedges’ *g* = 0.74, 95% CI: -0.13–1.60), AE (Hedges’ *g* = 1.69, 95% CI: -0.02–3.39), and exergaming (Hedges’ *g* = 0.03, 95% CI: -1.11–1.16) did not show statistically significant effects.

Measurement instrument was not a significant moderator of OCS (*QM*(3) = 1.79, *p* = .617). Significant effects were observed for TGMD (Hedges’ *g* = 1.19, 95% CI: 0.43–1.95) and BOT (Hedges’ *g* = 1.91, 95% CI: 0.58–3.25), whereas PDMS-2 (Hedges’ *g* = 1.00, 95% CI: -0.75–2.75) and MABC-2 (Hedges’ *g* = 0.82, 95% CI: -0.10–1.74) did not reach statistical significance.

Age was not a significant moderator of OCS (*QM*(1) = 0.38, *p* = .535). Significant effects were observed for 3–7 years (Hedges’ *g* = 0.94, 95% CI: 0.14–1.74) and 7–12 years (Hedges’ *g* = 1.25, 95% CI: 0.66–1.85).

#### 3.5.2 LMS subgroup analysis

Subgroup analyses suggested that exercise interventions produced significant improvements in LMS across most intervention conditions (**Figure 6**). Both intervention duration and intervention frequency significantly moderated the effects, indicating that these dosage-related factors may influence locomotor outcomes.

**Figure 6:**
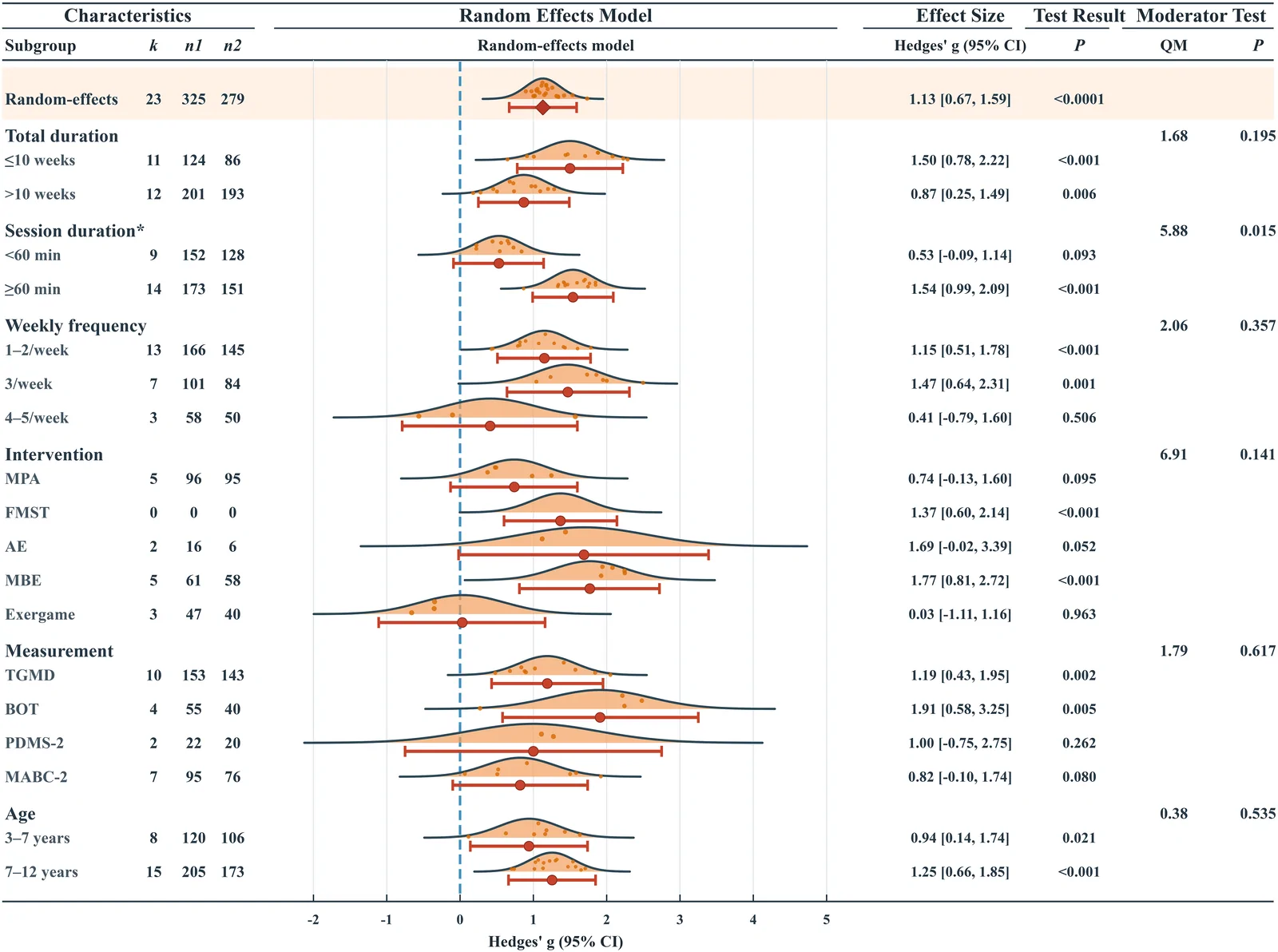
Subgroup analysis of LMS. Note. n1 = number of participants in the intervention group; n2 = number of participants in the control group.

Intervention frequency was a significant moderator of LMS (*QM*(2) = 8.06, *p* = .018). Significant effects were observed for 1–2 sessions/week (Hedges’ *g* = 0.98, 95% CI: 0.59–1.37) and 3 sessions/week (Hedges’ *g* = 1.72, 95% CI: 1.23–2.21), with the latter showing the largest effect. In contrast, the 4–5 sessions/week subgroup did not reach statistical significance (Hedges’ *g* = 0.58, 95% CI: -0.16–1.32).

Intervention duration significantly moderated the intervention effect on LMS (*QM*(1) = 5.84, *p* = .016). Both ≤ 10 weeks (Hedges’ *g* = 1.72, 95% CI: 1.19–2.25) and > 10 weeks (Hedges’ *g* = 0.96, 95% CI: 0.65–1.27) programs showed significant improvements, with a larger effect observed in interventions lasting ≤ 10 weeks.

Exercise session duration did not significantly moderate locomotor outcomes (*QM*(1) = 0.64, *p* = .424). Significant effects were observed for both < 60 min (Hedges’ *g* = 1.01, 95% CI: 0.45–1.57) and ≥ 60 min (Hedges’ *g* = 1.29, 95% CI: 0.89–1.69) sessions.

Intervention type did not significantly explain between-subgroup variation in LMS (*QM*(4) = 0.90, *p* = .925). Significant within-subgroup effects were observed for MPA (Hedges’ *g* = 1.24, 95% CI: 0.55–1.92), FMST (Hedges’ *g* = 1.08, 95% CI: 0.53–1.64), AE (Hedges’ *g* = 1.69, 95% CI: 0.26–3.11), and MBE (Hedges’ *g* = 1.49, 95% CI: 0.40–2.57). Exergaming showed a positive effect but did not reach statistical significance (Hedges’ *g* = 1.16, 95% CI: -0.26–2.57).

Measurement instrument was not a significant moderator of LMS (*QM*(3) = 0.59, *p* = .744). Significant effects were observed for TGMD (Hedges’ *g* = 1.23, 95% CI: 0.81–1.65), BOT (Hedges’ *g* = 1.06, 95% CI: 0.45–1.68), and the Agility T-test (Hedges’ *g* = 2.71, 95% CI: 0.96–4.46), whereas PDMS-2 did not reach statistical significance (Hedges’ *g* = 0.84, 95% CI: -0.16–1.84).

Age was not a significant moderator of LMS (*QM*(1) = 0.96, *p* = .328). Significant effects were observed for 3–7 years (Hedges’ *g* = 0.99, 95% CI: 0.47–1.51) and 7–12 years (Hedges’ *g* = 1.32, 95% CI: 0.91–1.73).

#### 3.5.3 SS subgroup analysis

Subgroup analyses showed that exercise interventions significantly improved SS across different intervention parameters (**Figure 7**). However, none of the examined moderators, including intervention frequency, intervention duration, exercise session duration, intervention type, and measurement instrument, significantly explained between-group differences.

**Figure 7:**
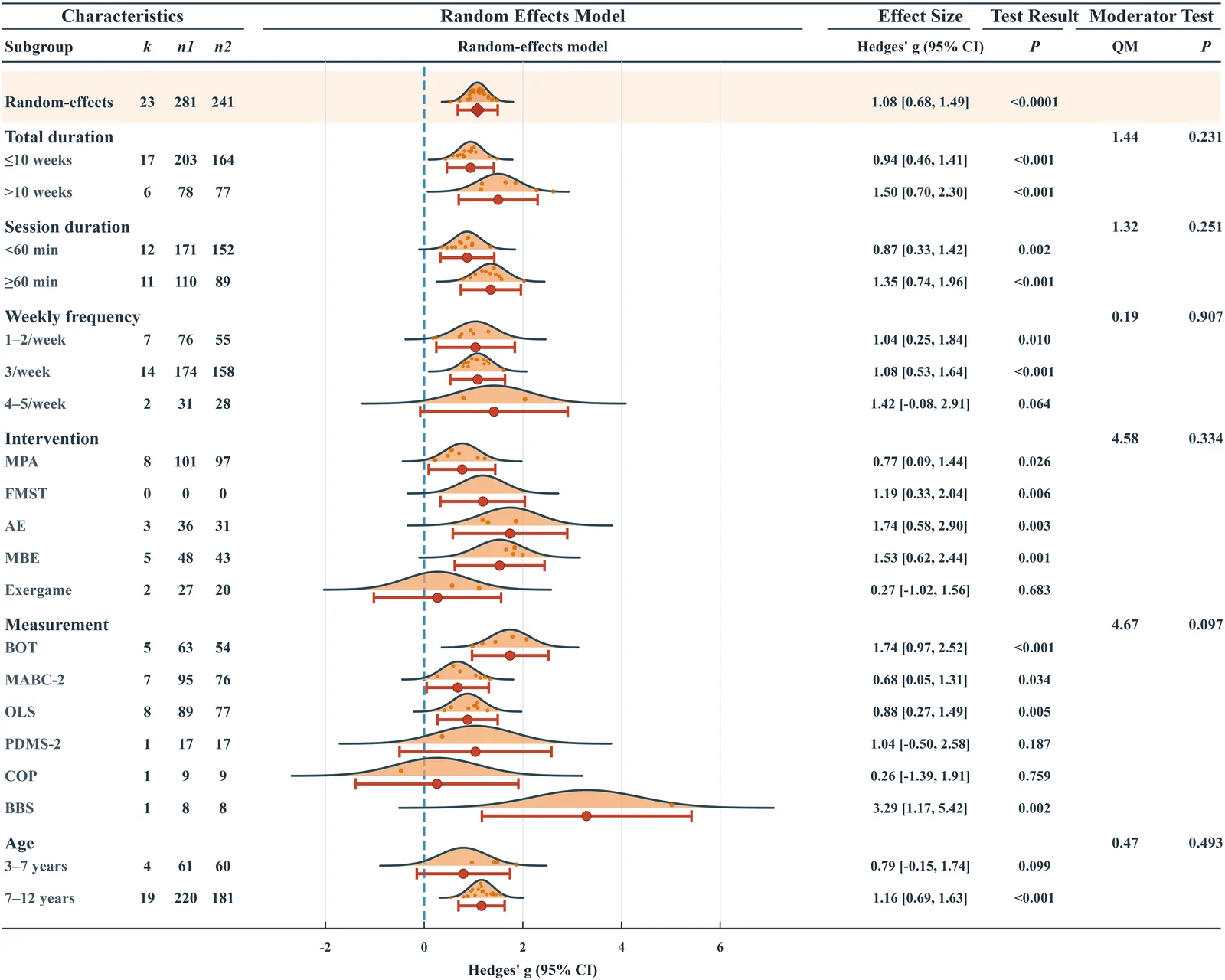
Subgroup analysis of SS. Note. n1 = number of participants in the intervention group; n2 = number of participants in the control group.

Intervention frequency was not a significant moderator of SS (*QM*(2) = 0.19, *p* = .906). Significant effects were observed for 1–2 sessions/week (Hedges’ *g* = 1.04, 95% CI: 0.25–1.84) and 3 sessions/week (Hedges’ *g* = 1.08, 95% CI: 0.53–1.64), whereas the 4–5 sessions/week subgroup did not reach statistical significance (Hedges’ *g* = 1.42, 95% CI: -0.08–2.91).

Intervention duration did not significantly moderate stability outcomes (*QM*(1) = 1.44, *p* = .231). Nevertheless, significant improvements were observed in both ≤ 10 weeks (Hedges’ *g* = 0.94, 95% CI: 0.46–1.41) and > 10 weeks (Hedges’ *g* = 1.50, 95% CI: 0.70–2.30) intervention programs.

Exercise session duration was not a significant moderator of SS (*QM*(1) = 1.32, *p* = .251). Significant effects were observed for both < 60 min (Hedges’ *g* = 0.87, 95% CI: 0.33–1.42) and ≥ 60 min sessions (Hedges’ *g* = 1.35, 95% CI: 0.74–1.96).

Intervention type did not significantly explain between-subgroup variation in SS (*QM*(4) = 4.58, *p* = .334). Significant within-subgroup effects were observed for MPA (Hedges’ *g* = 0.77, 95% CI: 0.09–1.44), FMST (Hedges’ *g* = 1.19, 95% CI: 0.33–2.04), AE (Hedges’ *g* = 1.74, 95% CI: 0.58–2.90), and MBE (Hedges’ *g* = 1.53, 95% CI: 0.62–2.44). In contrast, exergaming did not reach statistical significance (Hedges’ *g* = 0.27, 95% CI: -1.02–1.56).

Measurement instrument was not a significant moderator of SS (*QM*(5) = 4.67, *p* = .097). Significant effects were observed for BOT (Hedges’ *g* = 1.74, 95% CI: 0.97–2.52), MABC-2 (Hedges’ *g* = 0.68, 95% CI: 0.05–1.31), OLS (Hedges’ *g* = 0.88, 95% CI: 0.27–1.49), and BBS (Hedges’ *g* = 3.29, 95% CI: 1.17–5.42). However, PDMS-2 (Hedges’ *g* = 1.04, 95% CI: -0.50–2.58) and COP (Hedges’ *g* = 0.26, 95% CI: -1.39–1.91) did not reach statistical significance.

Age was not a significant moderator of SS (*QM*(1) = 0.47, *p* = .493). A significant effect was observed for 7–12 years (Hedges’ *g* = 1.16, 95% CI: 0.69–1.63), whereas 3–7 years (Hedges’ *g* = 0.79, 95% CI: -0.15–1.74) did not reach statistical significance.

Across the subgroup analyses, several subgroup estimates did not reach statistical significance, with their 95% CIs crossing zero. As shown in **Figures 5**, **6**, **7**, some of these subgroups included relatively few studies and participants, and the resulting estimates were accompanied by relatively wide confidence intervals. The uncertainty surrounding these estimates may therefore be partly related to the limited number of studies and participants available in these subgroups. Accordingly, these findings should be interpreted with caution and should not be taken as definitive evidence of an absence of intervention effects.

### 3.6 Meta-regression analyses

To further explore potential sources of between-study heterogeneity, meta-regression analyses were conducted to examine whether exercise-dose variables, including exercise session duration, intervention duration, total intervention dose, and intervention frequency, were associated with improvements in FMS. Total intervention dose was calculated as intervention duration × intervention frequency × exercise session duration and was scaled in units of 100 min for the meta-regression analyses.

For OCS, exercise session duration was a significant positive predictor of effect size (*β* = 0.05, 95% CI: 0.02–0.08, *z* = 3.56, *p* <.001; *R*² = 59.64%), indicating that longer individual exercise sessions were associated with greater improvements in OCS. In contrast, intervention duration, total intervention dose, and intervention frequency were not significant predictors (*p = .293, p = .979, and p = .506*, respectively; **Figure 8**).

**Figure 8:**
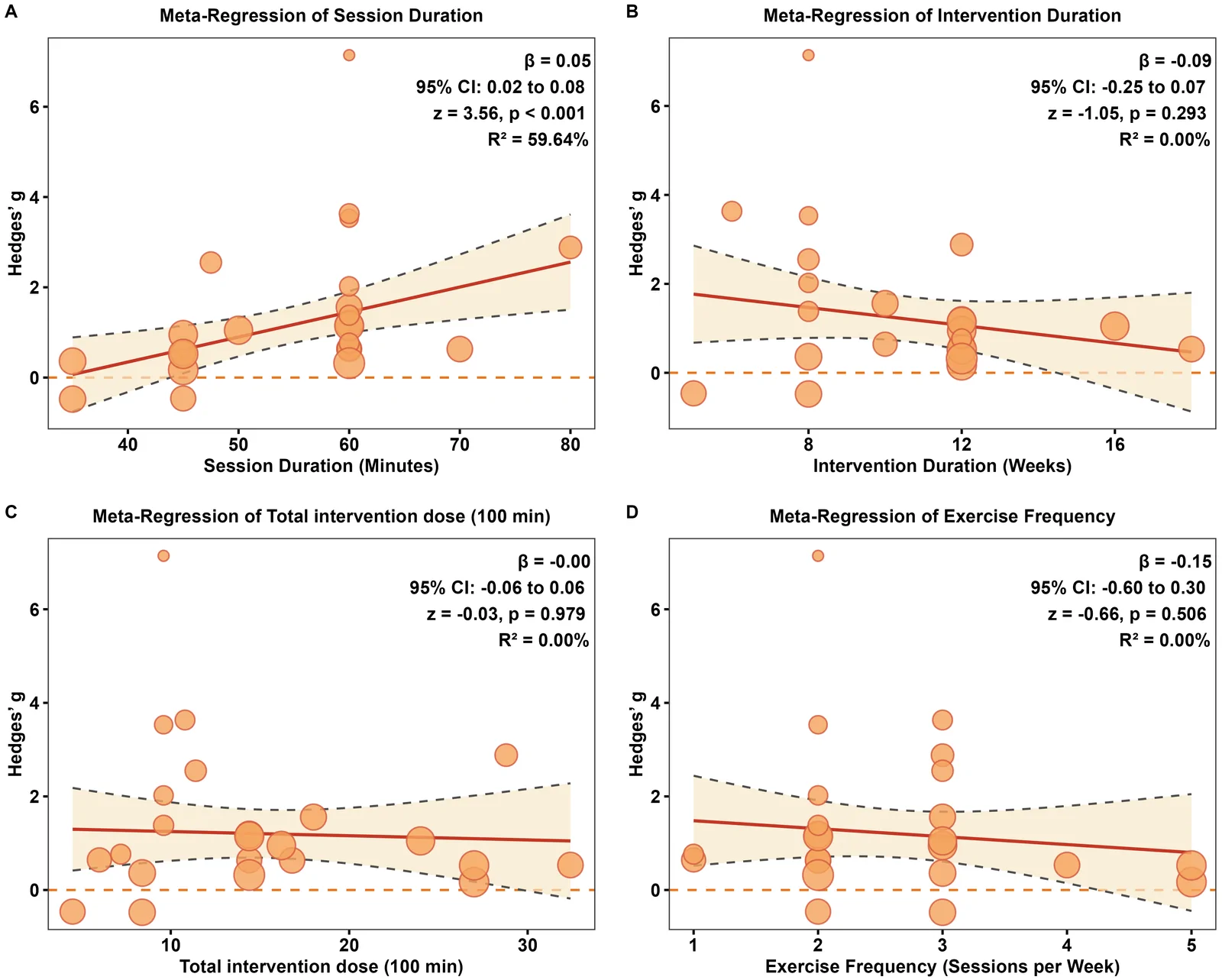
Meta-regression analysis of OCS: **(A)** session duration; **(B)** intervention duration; **(C)** total intervention dose, expressed in 100-min units; and **(D)** exercise frequency.

For LMS, none of the examined exercise-dose variables significantly predicted effect sizes. Intervention duration showed a negative association that approached statistical significance (*β* = -0.11, 95% CI: -0.22–0.00, *z* = -1.95, *p* = .051; *R*² = 24.42%), whereas exercise session duration, total intervention dose, and intervention frequency were not significant predictors (*p = .126, p = .925, and p = .955*, respectively; **Figure 9**).

**Figure 9:**
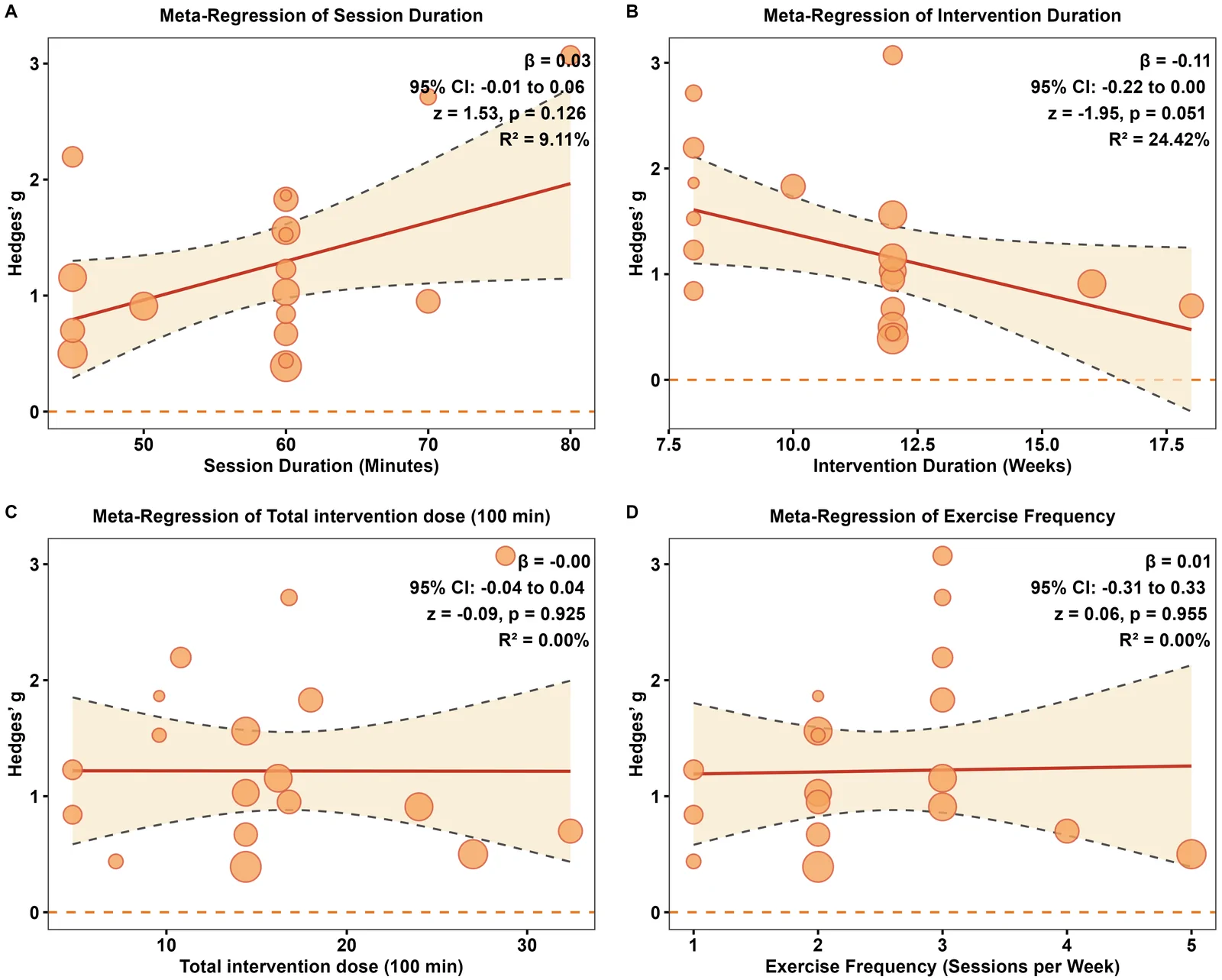
Meta-regression analysis of LMS: **(A)** session duration; **(B)** intervention duration; **(C)** total intervention dose, expressed in 100-min units; and **(D)** exercise frequency.

For SS, none of the examined exercise-dose variables were significant predictors of effect sizes. Exercise session duration showed a positive but non-significant association with stability outcomes (*p* = .116), whereas intervention duration, total intervention dose, and intervention frequency were also not significant predictors (*p = .189, p = .174, and p = .752*, respectively; **Figure 10**).

**Figure 10:**
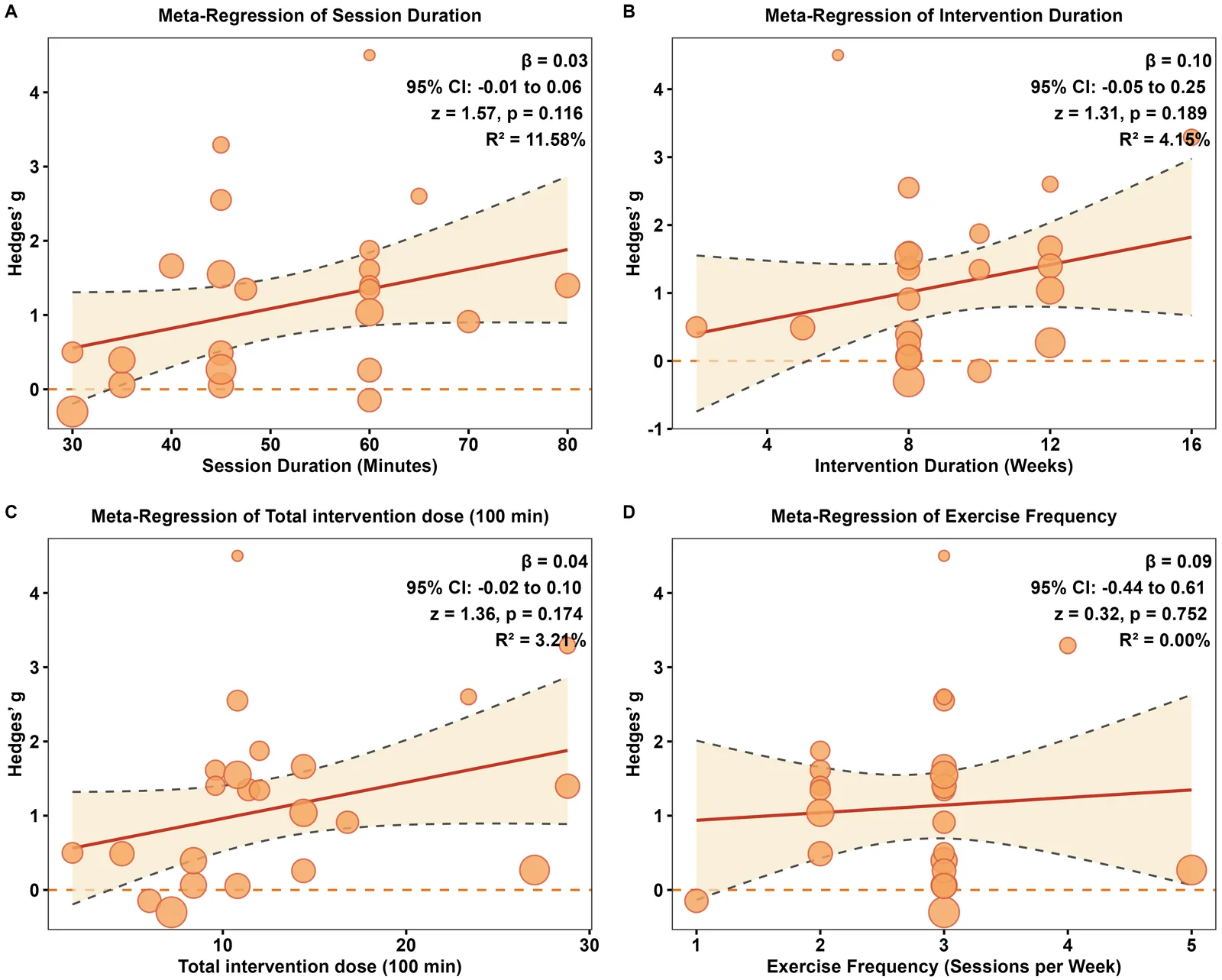
Meta-regression analysis of SS: **(A)** session duration; **(B)** intervention duration; **(C)** total intervention dose, expressed in 100-min units; and **(D)** exercise frequency.

### 3.7 Sensitivity analysis

Leave-one-out sensitivity analyses (**Supplementary Figure 1**-**S3**) confirmed the robustness of the meta-analysis findings. For OCS, the pooled effect estimates ranged from Hedges’ *g* = 0.99 to 1.19 after sequentially removing individual studies, and all 95% confidence intervals remained above the zero-effect line. The heterogeneity remained substantial, with *I*² values ranging from 77.84% to 84.74%. For LMS, pooled estimates ranged from Hedges’ *g* = 1.08 to 1.26, with moderate heterogeneity (*I*² = 39.22%–57.69%), and the direction and significance of the overall effects remained unchanged. For SS, pooled effects ranged from Hedges’ *g* = 0.97 to 1.15, with *I*² values ranging from 69.90% to 77.64%.To further assess the stability of the findings, studies with PEDro scores of 4–5 were excluded. For OCS, the pooled effect changed from Hedges’ g = 1.13 (95% CI: 0.67–1.59) to 1.35 (95% CI: 0.64–2.06). For LMS, the pooled effect changed from Hedges’ g = 1.19 (95% CI: 0.87–1.51) to 1.31 (95% CI: 0.93–1.69). For SS, the pooled effect changed from Hedges’ g = 1.08 (95% CI: 0.68–1.49) to 1.05 (95% CI: 0.64–1.45) (**Supplementary Figure 4**-**S6**). All pooled effects remained statistically significant after exclusion. Overall, the direction and significance of the pooled effects remained unchanged across both sensitivity analyses, supporting the stability of the findings.

### 3.8 Publication bias analysis

To assess potential publication bias, funnel plots were constructed for the OCS, LMS, and SS groups (**Supplementary Figures 7**-**S12**). Visual inspection showed some asymmetry across the three outcomes. Egger’s tests were significant for OCS (*z* = 5.35, *p* <.001), LMS (*z* = 2.61, *p* = .009), and SS (*z* = 5.29, *p* <.001), indicating potential small-study effects. Several studies with lower precision also showed relatively large positive effect sizes, suggesting that smaller studies may have reported larger intervention effects. However, the trim-and-fill analysis did not identify any missing studies for OCS, LMS, or SS (k = 0), and the pooled estimates remained unchanged. These findings suggest that small-study effects may be present, but they did not substantially influence the pooled estimates. Given the funnel plot asymmetry and substantial heterogeneity, the results should still be interpreted with caution.

## 4 Discussion

The updated meta-analysis synthesized evidence from 31 randomized controlled trials contributing 64 domain-specific effect-size estimates and demonstrated that exercise interventions effectively improved FMS in children and adolescents with ASD, including OCS, LMS, and SS. These findings are consistent with previous systematic reviews and meta-analyses reporting beneficial effects of exercise interventions on motor skill development in children with ASD (18, 20, 22). Furthermore, moderator analyses were conducted to explore whether different FMS domains showed distinct responses to intervention characteristics. The findings suggested domain-specific patterns in response to exercise dose. Improvements in OCS were associated with exercise session duration, with longer sessions (≥ 60 min) showing larger effects. LMS improvements were associated with intervention duration and frequency, with relatively larger effects observed for shorter intervention periods (≤ 10 weeks) and three sessions per week. In contrast, although SS was significantly improved following exercise interventions, none of the examined intervention characteristics showed significant moderating effects.

### 4.1 Effects of exercise interventions on OCS

#### 4.1.1 Potential mechanisms

The results of the meta-analysis demonstrate that exercise interventions produce significant improvements in OCS among children and adolescents with ASD (Hedges’ *g* = 1.13, 95% CI: 0.67–1.59), aligning with earlier evidence (18, 66). The observed improvements in OCS may be related to neurocognitive and motor-learning processes. On the one hand, exercise intervention may promote neuroplasticity and multisensory integration (67), potentially enhancing the efficiency of information processing across sensory pathways, including visual, vestibular, and proprioceptive systems. On the other hand, exercise intervention may also strengthen hand–eye coordination and executive functions (68, 69), thereby potentially contributing to improved movement regulation and control.

#### 4.1.2 Moderating effects of intervention characteristics

Regarding exercise session duration, the present study found that it may represent an important dosage factor influencing improvements in OCS. Subgroup analysis showed that interventions with exercise session duration of ≥ 60 minutes produced greater effects than those with durations of < 60 minutes. Furthermore, meta-regression analysis revealed a significant positive association between exercise session duration and OCS effect sizes (*p* <.001). According to motor learning theory, motor skill acquisition is facilitated by sufficient practice opportunities, repeated task engagement, and feedback-based adjustments (70). For children and adolescents with ASD, difficulties in motor coordination, motor planning, and motor control-related processing may be present (6), which may increase the need for repeated practice opportunities to facilitate the formation of stable motor patterns. Therefore, longer exercise session duration may provide more opportunities for movement attempts and repeated practice, thereby promoting the development of OCS. Furthermore, from a neuroplasticity perspective, repetitive motor training may induce experience-dependent neuroplastic changes through sustained sensorimotor stimulation and support the formation and strengthening of training-related motor representations (71, 72).

However, longer exercise sessions do not necessarily provide greater benefits. Excessively prolonged sessions may increase fatigue levels and reduce sustained attention, engagement, and movement execution quality, particularly among children and adolescents with ASD. Therefore, future studies should consider multiple factors, including exercise intensity, task complexity, variation in training content, and individual characteristics, to further identify optimal dosage combinations for improving OCS.

Regarding intervention type, previous meta-analyses have suggested that AE may provide significant benefits for improving OCS in children with ASD (18). However, the present meta-analysis did not confirm this advantage. Although AE showed a relatively large effect size (Hedges’ *g* = 1.69), the confidence interval crossed zero (95% CI: -.02–3.39), indicating that the effect did not reach statistical significance. Moreover, intervention type did not significantly explain between-subgroup differences in OCS improvements (*QM*(4) = 6.91, *p* = .141), suggesting that no specific exercise modality demonstrated a confirmed advantage for improving OCS in the present analysis.

In contrast, significant within-group effects were observed for FMST (Hedges’ *g* = 1.37, 95% CI:.60–2.14) and MBE (Hedges’ *g* = 1.77, 95% CI:.81–2.72). The beneficial effects of FMST may be attributed to its task-specific characteristics, as these interventions directly target object manipulation skills, including throwing, catching, and kicking, providing repeated opportunities to develop hand–eye coordination, movement planning, and motor control. MBE showed the largest effect size in the present analysis, which may be associated with its emphasis on postural control, body awareness, and attentional regulation. Through postural training, breathing control, and focused attention, MBE may enhance movement coordination and body awareness, thereby facilitating motor control and movement execution (51). Furthermore, MBE may improve responsiveness to external stimuli by strengthening sensory processing abilities (40). Sustained mind–body practice has also been suggested to promote functional connectivity and neuroplastic adaptations, potentially enhancing neural transmission efficiency involved in motor timing control and response regulation (73). However, these findings should be interpreted cautiously because exercise type did not significantly moderate OCS improvements, and the relatively larger effect observed for MBE does not indicate its superiority over other exercise modalities.

### 4.2 Effects of exercise interventions on LMS

#### 4.2.1 Potential mechanisms

The meta-analysis revealed that exercise interventions significantly improved LMS in children and adolescents with ASD (Hedges’ *g* = 1.19, 95% CI: 0.87–1.51), which is consistent with previous findings (74). LMS refers to the ability to perform whole-body movements, such as running, jumping, and climbing, which require the integration of lower-limb strength, coordination, and cardiorespiratory capacity. The observed improvements in LMS may be related to the combined effects of neurobiological adaptations, physical fitness enhancement, and motor coordination development. At the neural level, exercise may contribute to neuroplastic adaptations and improved functional connectivity, which may support the acquisition and retention of motor skills (75, 76). At the physiological level, exercise training may improve muscle strength and endurance in children with ASD (77), providing essential physical support for performing and sustaining locomotor movements. Furthermore, exercise may enhance motor coordination and movement control, which are important for the development of locomotor abilities in children with ASD (78).

#### 4.2.2 Moderating effects of intervention characteristics

Regarding intervention duration, the present study found that shorter intervention periods (≤ 10 weeks) resulted in greater improvements in LMS among children and adolescents with ASD compared with longer intervention periods (> 10 weeks). However, meta-regression analysis did not identify a significant association between intervention duration and effect sizes for LMS, indicating that the influence of intervention duration on LMS improvement requires further investigation. Consistent with our findings, Li et al. (79) reported that interventions lasting ≤ 10 weeks produced greater improvements in LMS than longer-duration interventions, suggesting that extending intervention duration does not necessarily result in additional motor skill benefits. This finding may be related to the challenges associated with maintaining children’s engagement and training participation during prolonged interventions. Previous research has indicated that repeated exposure to the same training content may reduce children’s interest and adherence, thereby attenuating intervention effects (80). For children and adolescents with ASD, who may experience difficulties in attention regulation and task switching (81, 82), longer intervention periods may further increase challenges in maintaining active participation and sustained attention, potentially affecting training quality and motor skill acquisition. Therefore, for LMS training in children and adolescents with ASD, appropriately controlling intervention duration and incorporating task variation and enjoyable activities to maintain engagement may be more beneficial than simply extending the intervention period.

The present study found that intervention frequency significantly moderated improvements in LMS, with three sessions per week showing the largest effect size compared with the 1–2 and 4–5 sessions/week categories. However, meta-regression analysis did not reveal a significant linear association between intervention frequency and LMS effect sizes, suggesting that differences among intervention frequency categories may be influenced by sample characteristics, intervention content, and other dosage-related factors. Lower intervention frequency may provide insufficiently consistent motor stimulation, limiting the accumulation of motor experience and consolidation of learned skills, thereby reducing improvements in LMS. In contrast, although higher intervention frequency increases opportunities for exercise exposure, excessively frequent sessions may increase physical and psychological demands in children and adolescents with ASD (83), potentially reducing engagement and movement quality during training.

### 4.3 Effects of exercise interventions on SS

#### 4.3.1 Potential mechanisms

The meta-analysis findings of this study indicate that exercise interventions significantly improve SS in children with ASD (Hedges’ *g* = 1.08, 95% CI: 0.68–1.49), which is consistent with previous research findings (84). The observed improvements in SS may be related to adaptive changes in sensory integration processes. Children with ASD often exhibit difficulties in processing and integrating visual, vestibular, and proprioceptive information (85). Regular exercise training may contribute to improved sensory processing through repeated sensorimotor experiences, enhanced proprioceptive input, and more efficient integration across multisensory pathways (86, 87). These neuroadaptive changes may support better coordination between sensory systems and provide a potential foundation for improved motor control and postural stability.

#### 4.3.2 Moderating effects of intervention characteristics

None of the examined factors—including intervention duration, intervention frequency, exercise session duration, intervention type, and measurement instrument—significantly moderated improvements in SS, suggesting that improvements in SS may not depend on a single intervention characteristic but may result from the combined effects of multiple training components. SS involve complex processes, including postural control, sensory integration, and neuromuscular coordination, which may be influenced by various factors during exercise training. In addition, variations in intervention protocols and the limited number of available studies may have reduced the ability to detect potential moderating effects. Future studies with larger samples and standardized intervention designs are needed to further clarify the factors influencing improvements in SS.

### 4.4 Clinical implications

The findings of this study may provide several implications for clinical and educational practice. Exercise interventions should be designed according to specific motor domains rather than applying a uniform approach for all FMS outcomes. Based on the subgroup findings, these domain specific dosage patterns may provide practical guidance for clinical and educational practice. For OCS, exercise programs combining an intervention duration of ≤10 weeks, a single-session duration of ≥60 minutes, three sessions per week, and MBE or FMST may be considered as potential strategies, with training emphasizing object-control activities such as throwing, catching, kicking, and other tasks requiring coordinated interaction with objects. For LMS, programs incorporating an intervention duration of ≤10 weeks, a single-session duration of ≥60 minutes, three sessions per week, and AE may be considered, with a focus on improving lower-limb strength, coordination, and endurance. For SS, interventions involving an intervention duration of >10 weeks, a single-session duration of ≥60 minutes, 1–2 sessions per week, and AE or MBE may represent potential approaches, with training focusing on balance, postural control, and body stability.

However, these recommendations should be interpreted with caution because they are based on subgroup analyses and do not represent optimal exercise prescriptions. Clinicians and special education practitioners should further adapt these parameters according to individual characteristics, such as age, baseline motor abilities, ASD severity, motivation, exercise tolerance, and available resources.

### 4.5 Limitations and future directions

Several limitations should be considered. First, although this study included 31 randomized controlled trials contributing 64 domain-specific effect-size estimates, the number of available studies was limited, and some subgroup analyses were based on a small number of studies, which may have affected the stability of moderator analyses. Second, heterogeneity existed across studies in participant characteristics, intervention protocols, and outcome measures. Important ASD-related characteristics, including ASD subtypes, ASD severity stratification, basic motor ability levels, intellectual ability, and comorbid conditions, were not consistently reported, limiting the interpretation of individual differences in response to exercise interventions. Third, methodological limitations should be noted. Due to the nature of exercise interventions, blinding of participants and therapists is difficult to implement. In addition, incomplete reporting of allocation concealment and assessor blinding in some studies may have increased the risk of bias. Fourth, potential small-study effects should be considered. The observed asymmetry may partly be related to the small sample sizes of some included studies. Effect estimates from small samples are often less stable and more easily influenced by extreme values. Finally, most studies did not include long-term follow-up assessments, limiting the evaluation of whether improvements in FMS were maintained over time. Future studies should include larger samples, provide more detailed participant information, adopt standardized intervention protocols, and include longer follow-up periods to further strengthen the evidence regarding exercise interventions for children and adolescents with ASD.

## 5 Conclusions

This meta-analysis included 31 randomized controlled trials and demonstrated that exercise interventions significantly improved FMS, including OCS, LMS, and SS, in children and adolescents with ASD. Further analyses indicated that different motor domains may exhibit distinct responses to intervention characteristics. Specifically, longer exercise session durations were associated with greater improvements in OCS, and meta-regression analyses further identified a significant positive association between session duration and OCS effects, indicating a potential dose–response relationship. In contrast, LMS showed greater effects with shorter intervention durations and an intervention frequency of three sessions per week, although these subgroup findings were not confirmed by significant dose–response associations. No significant moderating effects of intervention characteristics were identified for SS, suggesting that exercise interventions should be tailored to specific motor domains rather than applying a uniform approach across all FMS outcomes. Future research should further investigate individualized exercise strategies through well-designed trials with standardized intervention protocols.

## Data Availability

The original contributions presented in the study are included in the article/**Supplementary Material**. Further inquiries can be directed to the corresponding author/s.
