# Supplementary material for: Effects of exercise interventions on fundamental motor skills in children and adolescents with autism spectrum disorder: a meta-analysis with meta-regression analyses of randomized controlled trials
Source: Front Psychiatry. 2026 Sep 30;17:1954728. doi: 10.3389/fpsyt.2026.1954728 (PMC13661967; doi:10.3389/fpsyt.2026.1954728)
Supplement: Supplementary file 1 [file SupplementaryFile1.docx]

Detailed Literature Search Strategy

Search Strategy

| **Database** | **Search strategy** | **Results** |
| --- | --- | --- |
| **PubMed** | ("Autism Spectrum Disorder"[MeSH Terms] OR "autism spectrum disorder*"[tiab] OR ASD[tiab] OR "autistic disorder*"[tiab] OR "autistic spectrum disorder*"[tiab] OR "disorder, autistic spectrum"[tiab] OR Asperger*[tiab] OR "Asperger disorder*"[tiab] OR "Asperger syndrome*"[tiab] OR "Asperger disease*"[tiab] OR "Asperger's disorder*"[tiab] OR "Asperger's syndrome*"[tiab] OR "childhood disintegrative disorder"[tiab] OR CDD[tiab] OR "Kanner* syndrome"[tiab] OR "infantile autism"[tiab] OR "early infantile autism"[tiab] OR "autism, infantile"[tiab] OR "autism, early infantile"[tiab]) AND ("Exercise"[MeSH Terms] OR "exercise"[tiab] OR" physical activit*"[tiab] OR "exercise therap*"[tiab] OR "motor activit*"[tiab] OR "physical training"[tiab] OR "fitness training"[tiab] OR"movement therap*"[tiab] OR rehabilitation[tiab] OR sports[tiab] OR "aerobic exercise"[tiab] OR "resistance training"[tiab] OR "strength training"[tiab] OR "endurance training"[tiab] OR "balance training"[tiab] OR swimming[tiab] OR running[tiab] OR cycling[tiab] OR "jump rope"[tiab] OR "ball game*"[tiab] OR basketball[tiab] OR soccer[tiab] OR football[tiab] OR volleyball[tiab] OR "table tennis"[tiab] OR "martial art*"[tiab] OR judo[tiab] OR karate[tiab] OR taekwondo[tiab] OR "kung fu"[tiab] OR dance[tiab] OR yoga[tiab] OR pilates[tiab] OR "tai chi"[tiab] OR qigong[tiab] OR baduanjin[tiab] OR wuqinxi[tiab] OR yijinjing[tiab] OR exergam*[tiab] OR "mind-body exercise"[tiab]) AND ("Motor Skills"[MeSH Terms] OR "fundamental movement skill*"[tiab] OR "gross motor skill*"[tiab] OR "gross motor"[tiab] OR "movement skill*"[tiab] OR "motor proficiency"[tiab] OR "motor performance"[tiab] OR "motor coordination"[tiab] OR "stability skill*"[tiab] OR "locomotor skill*"[tiab] OR locomotor[tiab] OR "object control skill*"[tiab] OR "ball skill*"[tiab] OR balance[tiab] OR "postural balance"[tiab] OR "static balance"[tiab] OR "dynamic balance"[tiab]) AND (child*[tiab] OR adolescen*[tiab] OR pediatric*[tiab] OR paediatric*[tiab] OR preschool*[tiab] OR "schoolage*"[tiab] OR youth[tiab] OR teen*[tiab]) | 181 |
| **Web of Science** | TS = ("autism spectrum disorder*" OR ASD OR "autistic disorder*" OR "autistic spectrum disorder*" OR "disorder, autistic spectrum" OR Asperger* OR "Asperger disorder*" OR "Asperger syndrome*" OR "Asperger disease*" OR "Asperger's disorder*" OR "Asperger's syndrome*" OR "childhood disintegrative disorder" OR CDD OR "Kanner* syndrome" OR "infantile autism" OR "early infantile autism" OR "autism, infantile" OR "autism, early infantile") AND TS = ("exercise" OR "physical activit*" OR "exercise therap*" OR "motor activit*" OR "physical training" OR "fitness training" OR "movement therap*" OR rehabilitation OR sports OR "aerobic exercise" OR "resistance training" OR "strength training" OR "endurance training" OR "balance training" OR swimming OR running OR cycling OR "jump rope" OR "ball game*" OR basketball OR soccer OR football OR volleyball OR "table tennis" OR "martial art*" OR judo OR karate OR taekwondo OR "kung fu" OR dance OR yoga OR pilates OR "tai chi" OR qigong OR baduanjin OR wuqinxi OR yijinjing OR exergam* OR "mind-body exercise") AND TS = ("motor skills" OR "fundamental movement skill*" OR "gross motor skill*" OR "gross motor" OR "movement skill*" OR "motor proficiency" OR "motor performance" OR "motor coordination" OR "stability skill*" OR "locomotor skill*" OR locomotor OR "object control skill*" OR "ball skill*" OR balance OR "postural balance" OR "static balance" OR "dynamic balance") AND TS = (child* OR adolescen* OR pediatric* OR paediatric* OR preschool* OR "school-age*" OR youth OR teen*) | 643 |
| **Embase** | ('autism spectrum disorder'/exp OR 'autism spectrum disorder*':ti,ab OR asd:ti,ab OR 'autistic disorder*':ti,ab OR 'autistic spectrum disorder*':ti,ab OR 'disorder, autistic spectrum':ti,ab OR asperger*:ti,ab OR 'asperger disorder*':ti,ab OR 'asperger syndrome*':ti,ab OR 'asperger disease*':ti,ab OR 'aspergers disorder*':ti,ab OR 'aspergers syndrome*':ti,ab OR 'childhood disintegrative disorder':ti,ab OR cdd:ti,ab OR 'kanner* syndrome':ti,ab OR 'infantile autism':ti,ab OR 'early infantile autism':ti,ab OR 'autism, infantile':ti,ab OR 'autism, early infantile':ti,ab) AND ('exercise'/exp OR exercise:ti,ab OR 'physical activit*':ti,ab OR 'exercise therap*':ti,ab OR 'motor activit*':ti,ab OR 'physical training':ti,ab OR 'fitness training':ti,ab OR 'movement therap*':ti,ab OR rehabilitation:ti,ab OR sports:ti,ab OR 'aerobic exercise':ti,ab OR 'resistance training':ti,ab OR 'strength training':ti,ab OR 'endurance training':ti,ab OR 'balance training':ti,ab OR swimming:ti,ab OR running:ti,ab OR cycling:ti,ab OR 'jump rope':ti,ab OR 'ball game*':ti,ab OR basketball:ti,ab OR soccer:ti,ab OR football:ti,ab OR volleyball:ti,ab OR 'table tennis':ti,ab OR 'martial art*':ti,ab OR judo:ti,ab OR karate:ti,ab OR taekwondo:ti,ab OR 'kung fu':ti,ab OR dance:ti,ab OR yoga:ti,ab OR pilates:ti,ab OR 'tai chi':ti,ab OR qigong:ti,ab OR baduanjin:ti,ab OR wuqinxi:ti,ab OR yijinjing:ti,ab OR exergam*:ti,ab OR 'mind-body exercise':ti,ab) AND ('motor skill'/exp OR 'fundamental movement skill*':ti,ab OR 'gross motor skill*':ti,ab OR 'gross motor':ti,ab OR 'movement skill*':ti,ab OR 'motor proficiency':ti,ab OR 'motor performance':ti,ab OR 'motor coordination':ti,ab OR 'stability skill*':ti,ab OR 'locomotor skill*':ti,ab OR locomotor:ti,ab OR 'object control skill*':ti,ab OR 'ball skill*':ti,ab OR balance:ti,ab OR 'postural balance':ti,ab OR 'static balance':ti,ab OR 'dynamic balance':ti,ab) AND (child*:ti,ab OR adolescen*:ti,ab OR pediatric*:ti,ab OR paediatric*:ti,ab OR preschool*:ti,ab OR 'school-age*':ti,ab OR youth:ti,ab OR teen*:ti,ab) | 407 |
| **Cochrane** | (MeSH descriptor: [Autism Spectrum Disorder] explode all trees OR (Disorder, Autistic Spectrum):ti,ab,kw OR (Autistic):ti,ab,kw OR (Autistic Spectrum Disorder):ti,ab,kw OR (Autistic Spectrum Disorders):ti,ab,kw OR (ASD):ti,ab,kw OR "childhood disintegrative disorder":ti,ab,kw) AND (MeSH descriptor: [Child] explode all trees OR Child:ti,ab,kw OR adolescent:ti,ab,kw OR pediatric:ti,ab,kw OR preschool:ti,ab,kw OR teenager:ti,ab,kw) AND (MeSH descriptor: [Motor Skills] explode all trees OR "Motor Skills":ti,ab,kw OR "Motor Skill":ti,ab,kw OR "fundamental movement skill":ti,ab,kw OR "gross motor skill":ti,ab,kw OR "object control skill":ti,ab,kw OR "postural balance":ti,ab,kw OR balance:ti,ab,kw OR "motor performance":ti,ab,kw OR "motor function":ti,ab,kw OR "motor proficiency":ti,ab,kw) AND (MeSH descriptor: [Exercise] explode all trees OR MeSH descriptor: [Sports] explode all trees OR MeSH descriptor: [Yoga] explode all trees OR MeSH descriptor: [Tai Ji] explode all trees OR exercise:ti,ab,kw OR "physical activity":ti,ab,kw OR "physical training":ti,ab,kw OR "aerobic exercise":ti,ab,kw OR "resistance training":ti,ab,kw OR sport:ti,ab,kw OR "ball games":ti,ab,kw OR "balance training":ti,ab,kw OR "strength training":ti,ab,kw OR "movement therapy":ti,ab,kw OR taijiquan:ti,ab,kw OR "Tai Chi":ti,ab,kw) | 125 |

**Supplementary Figures**

Supplementary Figure S1. Leave-one-out sensitivity analysis for OCS.

Supplementary Figure S2. Leave-one-out sensitivity analysis for LMS.

Supplementary Figure S3. Leave-one-out sensitivity analysis for SS.

Supplementary Figure S4. Sensitivity analysis for OCS after excluding studies with PEDro scores of 4–5.

Supplementary Figure S5. Sensitivity analysis for LMS after excluding studies with PEDro scores of 4–5.

Supplementary Figure S6. Sensitivity analysis for SS after excluding studies with PEDro scores of 4–5.

Supplementary Figure S7. Funnel plot for OCS.

Supplementary Figure S8. Funnel plot for LMS.

Supplementary Figure S9. Funnel plot for SS.

Supplementary Figure S10. Trim-and-fill funnel plot for OCS.

Supplementary Figure S11. Trim-and-fill funnel plot for LMS.

Supplementary Figure S12. Trim-and-fill funnel plot for SS.
